# The Role of the Membrane Potential in Chondrocyte Volume Regulation

**DOI:** 10.1002/jcp.22646

**Published:** 2011-02-15

**Authors:** Rebecca Lewis, Katie E Asplin, Gareth Bruce, Caroline Dart, Ali Mobasheri, Richard Barrett-Jolley

**Affiliations:** 1 Department of Musculoskeletal Biology, Institute of Aging and Chronic Disease, Faculty of Health and Life Sciences, University of LiverpoolLiverpool, UK; 2 Musculoskeletal Research Group, Division of Veterinary Medicine, Faculty of Medicine and Health Sciences, School of Veterinary Medicine and Science, University of NottinghamLoughborough, UK; 3 Institute of Membrane and Systems Biology, University of LeedsLeeds, UK; 4 Institute of Integrative Biology, Faculty of Health and Life Sciences, University of LiverpoolLiverpool, UK

## Abstract

Many cell types have significant negative resting membrane potentials (RMPs) resulting from the activity of potassium-selective and chloride-selective ion channels. In excitable cells, such as neurones, rapid changes in membrane permeability underlie the generation of action potentials. Chondrocytes have less negative RMPs and the role of the RMP is not clear. Here we examine the basis of the chondrocyte RMP and possible physiological benefits. We demonstrate that maintenance of the chondrocyte RMP involves gadolinium-sensitive cation channels. Pharmacological inhibition of these channels causes the RMP to become more negative (100 µM gadolinium: ΔV_m_ = −30 ± 4 mV). Analysis of the gadolinium-sensitive conductance reveals a high permeability to calcium ions (PCa/PNa ≈80) with little selectivity between monovalent ions; similar to that reported elsewhere for TRPV5. Detection of TRPV5 by PCR and immunohistochemistry and the sensitivity of the RMP to the TRPV5 inhibitor econazole (ΔV_m_ = −18 ± 3 mV) suggests that the RMP may be, in part, controlled by TRPV5. We investigated the physiological advantage of the relatively positive RMP using a mathematical model in which membrane stretch activates potassium channels allowing potassium efflux to oppose osmotic water uptake. At very negative RMP potassium efflux is negligible, but at more positive RMP it is sufficient to limit volume increase. In support of our model, cells clamped at −80 mV and challenged with a reduced osmotic potential swelled approximately twice as much as cells at +10 mV. The positive RMP may be a protective adaptation that allows chondrocytes to respond to the dramatic osmotic changes, with minimal changes in cell volume. J. Cell. Physiol. 226: 2979–2986, 2011. © 2011 Wiley-Liss, Inc.

Chondrocytes are the cells that produce, maintain, and degrade the extracellular matrix of articular cartilage in load-bearing joints (Huber et al., 2000; Archer and Francis-West, 2003). Their location, embedded in cartilage, means that these cells are subjected to significant dynamic loads during physical activity (Eckstein et al., 1999). The limb joints in a galloping horse, for example, will routinely experience compressive forces of 7,500 N (Setterbo et al., 2009). Contact pressures have been directly measured in human hip joints and are reported to be as high as 18 MPa (Hodge et al., 1986). Under pressure, cartilage exudes fluid (McCutchen, 1962) and thus decreases in volume. This involves changes in water content of the interstitial component of cartilage and consequent changes in extracellular osmotic potential (Mow et al., 1992, 1999; Sivan et al., 2006). Typical osmolarities for many mammalian cells are in the region of 300 mOsm. However, under load, the osmolarity of the extracellular matrix of cartilage is believed to be approximately 480 mOsm (Urban, 1994). More recently, osmolarities as high as 550 mOsm have been used to model the three-dimensional microenvironment of chondrocytes under load (Xu et al., 2010). This means that chondrocytes exist in a unique cellular environment. Changes in osmotic pressure are reversible upon relaxation (Mow et al., 1992; Urban, 1994) and so during normal usage chondrocytes will be cyclically exposed to both increasing and decreasing osmotic forces. Healthy chondrocytes are able to regulate their volume with remarkable resilience throughout these osmotic pressure cycles (Bush and Hall, 2001a). However, chondrocytes swell during the decreasing phases of the osmotic pressure cycle (Bush and Hall, 2001b) and are vulnerable to damage (Bush et al., 2005). Decrease in osmotic potential (increased water content) has been linked to the early onset of osteoarthritis (Stockwell, 1991) and loss of volume control has been specifically linked to chondrocyte death and the progression of osteoarthritis (Bush and Hall, 2003; Bush et al., 2005).

As a starting point to understanding how these specialized cells so effectively regulate their volume and the membrane ion fluxes involved, we decided to make a thorough examination of their resting membrane potential (RMP) properties in vitro. There have been a number of previous studies, which observe chondrocytes RMP in a range of species, but these have resulted in a very wide variety of values from −10.6 to −46 mV (Wright et al., 1992; Sugimoto et al., 1996; Clark et al., 2010; Funabashi et al., 2010b). We began by thoroughly examining the RMP of chondrocytes from larger mammals in vitro and found these to be much less negative than other cell types. Previous studies have investigated the role of potassium (Wilson et al., 2004) and chloride (Tsuga et al., 2002; Funabashi et al., 2010a) channels in the regulation of the RMP and so we examined the contribution of sodium and/or non-specific cation conductances. We then investigated what the physiological advantage of such positive RMPs is for chondrocytes.

## Methods

Canine cartilage was removed from stifle and elbow condyles of large, skeletally mature, bull terrier types euthanatized for unrelated clinical reasons. Bovine, ovine, and equine cartilage were sourced from a local abattoir. Chondrocytes were isolated as described previously (Mobasheri et al., 2005, 2007, 2010) with type II collagenase. To ensure preservation of the chondrocyte phenotype we used only cartilage slices (collagenase treated), freshly dissociated, first expansion and first passage cells only. RT-PCR experiments used first expansion chondrocytes. When isolated, chondrocyte doubling time was within 24 h confirming that these cells are viable and vital. Other cell types were prepared by their respective standard methods: rat dorsal root ganglion neurones (Bruce et al., 2009), hypothalamic pre-autonomic neurones (Barrett-Jolley et al., 2000; Womack et al., 2007), and aortic smooth muscle (Sampson et al., 2007).

### Electrophysiology

RMP were measured using whole-cell patch clamp in current clamp mode by three different amplifiers (Axon Axopatch 200a, 200b, Molecular Devices, Sunnyvale, CA Cairns Optoclamp, Faversham, UK). For RMP measurements we used a standard physiological saline for both intracellular and extracellular solutions of (in mM): 95 K-Gluconate, 26 KCl, 1 MgCl_2_, 5 BAPTA and 10 HEPES (pH 7.2 with KOH), and 140 NaCl, 5 KCl, 2 CaCl_2_, 1 MgCl_2_ and 10 HEPES (pH 7.4 with NaOH), respectively. For sharp electrode recording (NPI SEC-05LX amplifier), electrodes were filled with 1 or 2 M KCl and the extracellular solutions were as above. For the cell-volume experiments, osmolarity was increased by addition of 180 mM sucrose.

Measurement of gadolinium III (Gd) difference currents was made with the Axon Axopatch 200a amplifier. A ramp protocol was applied consisting of a 50-msec voltage step at 0 mV followed by a 4.5 sec linear ramp from −60 to +80 mV. This was repeated every 50 sec. Whole-cell currents were recorded in “methanesulfonate solutions,” containing: (in mM): 150 Na-methanesulfonate, 10 HEPES, 2 CaCl_2_ (pH 7.4 with NaOH) in the bath and 150 Na-methanesulfonate, 10 HEPES, 5 mM BAPTA (pH 7.4 with NaOH) in the pipette solution. Difference currents were obtained by subtraction of a ramp in the presence of 100 µM Gd from that run in vehicle control.

To study the relative whole-cell permeability of monovalent cations and calcium ions, we used bath solutions containing (in mM) 2 MgCl_2_, 10 HEPES and either 150 XCl (where X = Na, K, or Cs) or 30 mM CaCl_2_ and 105 mM NaCl (all pH to 7.4 with the relevant monovalent hydroxide). In these experiments, the patch pipette (intracellular) solution contained (in mM): 135 CsCl, 1 MgCl_2_, 10 Hepes, and 5 EGTA (pH 7.2 with NaOH). Permeability ratios were calculated using the equations of Voets et al. (2002), using the reversal potentials (V_rev_) of the Gd sensitive current–voltage ramps.For monovalent cations:


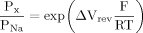
(1)

For calcium ions:


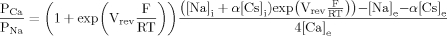
(2)

where


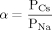
(3)

### Video imaging and switch clamp

Cells were voltage clamped with single sharp electrodes, under switch clamp (SEC 05LX, NPI) and simultaneously videoed with a Hitachi (KP-M3E/K CCD) camera attached to a Nikon Eclipse microscope magnification ∼1,000×.

### Analysis

Electrophysiological data were digitized and analyzed using the WinEDR and WinWCP programs (John Dempster, University of Strathclyde). Visual data were analyzed with ImageJ (Abramoff et al., 2004) and ANOVA performed with SPSS (SPSS, Inc., Chicago, IL), multiple comparisons assessed with Dunnett tests. *t*-Tests were performed with Minitab (Minitab Ltd, Coventry, UK). All values are quoted as mean ± SEM, with sample size = n. All membrane potentials are corrected for liquid junction potentials estimated using JPCalc (Barry and Lynch, 1991).

### Voltage-sensitive dye membrane potential measurements

To measure the membrane potential with optical dyes we used oxonol VI (Apell and Bersch, 1987; Wohlrab et al., 2001). Oxonol VI (100–300 nM) was added to the perfusion solution, for at least 15 min prior to commencement of recording. The optical system (Hitachi KP-M3E/K CCD camera attached to a Nikon Eclipse microscope, G-2A filter set, magnification ∼1,000×) was calibrated by measuring the average intensity in a chondrocyte, under perforated patch clamp at a range of membrane potentials. Our perforated patch-clamp methods have been described elsewhere (Davies et al., 2010). This intensity versus voltage calibration curve was then used to extrapolate membrane potentials of surrounding un-clamped chondrocytes.

### RT-PCR

Total RNA was extracted from canine chondrocytes by use of an RNeasy Mini Kit (Qiagen, Crawley, UK) according to manufacturer's instructions. Genomic DNA was eliminated using deoxyribonuclease I, Amplification Grade (Invitrogen, Paisley, UK). Isolated total RNA was then used as a template to create first-strand cDNA by Superscript II Reverse Transcriptase (Invitrogen). One microliter of this cDNA was used as a template for touchdown PCR using the primers in Table 1 (derived from murine transient receptor potential vanilloid channel subtype 5, TRPV5). PCR was performed with 35 cycles in total on a Techgene FTGene2D thermocycler (Techne, Stone, UK). The initial cycle consisted of a denaturation step at 92°C for 30 sec, an annealing step at 65°C for 30 sec, and an extension step of 72°C for 60 sec. The annealing step was decreased by 1°C with each cycle to a final temp of 56°C. PCR products (10 µl) were separated by electrophoresis on a 1.5% agarose gel (2 h at 80 mV) and the bands were visualized by Gel Red (Biotium, Cambridge, UK) staining using a UV transilluminator (BioRad, Hemel Hempstead, UK). Sequencing was performed by Beckman Coulter Genomics (Takeley, UK).

**Table 1:** Primer sequences for each of the proteins investigated

Protein	Primer pair sequences (5′–3′)	Size (bp)
TRPV5	Forward: GCCCCTAACATCTTCCCTCT	165
	Reverse: TGTCCATATTTCTTCCACTT	
GAPDH	Forward: CATCAACGGGAAGTCCATCT	429
	Reverse: GTGGAAGCAGGGATGATGTT	

GAPDH was used as a test for viable cDNA. Primers were purchased from Sigma-Aldrich, Poole, UK.

### Ion channel immunohistochemistry

Sections of canine cartilage were probed for channel expression by immunohistochemistry essentially as described previously (Mobasheri et al., 2005). Slides were deparaffinized in xylene for 20 min to remove embedding medium, washed in absolute ethanol for 3 min, gradually rehydrated in a series of alcohol baths (96%, 85%, and 50%) and then placed water for 5 min. Endogenous peroxidase activity was blocked for 1 h in a solution of 97% methanol, 3% hydrogen peroxide, and 0.01% sodium azide. The slides were then incubated for 1 h at room temperature in tris-buffered saline (TBS) containing 1% bovine serum albumin (BSA, protease-free) and 0.01% sodium azide to block non-specific antibody binding. Slides were incubated overnight at 4°C with rabbit polyclonal antibodies to TRPV5 (Abcam plc., Cambridge, UK). Antibodies were diluted (various dilutions ranging from 1:200 to 1:1,500) in TBS containing 1% BSA. After 24 h at 4°C, the slides were washed three times for 5 min each in TBS containing 0.05% Tween-20 (TBS-T) before incubation with horseradish peroxidase-labeled polymer conjugated to affinity-purified goat anti-rabbit immunoglobulins (code no. K4010; Dako UK Ltd, Ely, UK) for 30 min at room temperature. The sections were washed a further three times for 5 min in TBS-T before application of liquid DAB+ chromogen (3,3′-diaminobenzidine solution; DakoCytomation). The development of the brown-colored reaction was stopped by rinsing in TBS-T. The stained slides were immersed for 5 min in a bath of aqueous hematoxylin (code no. S3309; DakoCytomation) to counterstain cell nuclei. Finally, the slides were washed for 5 min in running water and dehydrated in a series of graded ethanol baths before being rinsed in three xylene baths and mounted in 1,3-diethyl-8-phenylxanthine (BDH Laboratories, Atherstone, UK). Control experiments were performed by omitting the primary antibody from the immunohistochemical procedure. Immunostained tissue sections were examined with a Nikon Eclipse 80i microscope. Photomicrographs were digitally captured using Nikon Digital Sight DS-5M camera and Nikon Eclipsenet image capture software.

## Results

### Initial measurements of chondrocyte RMP

**Fig. 1:**
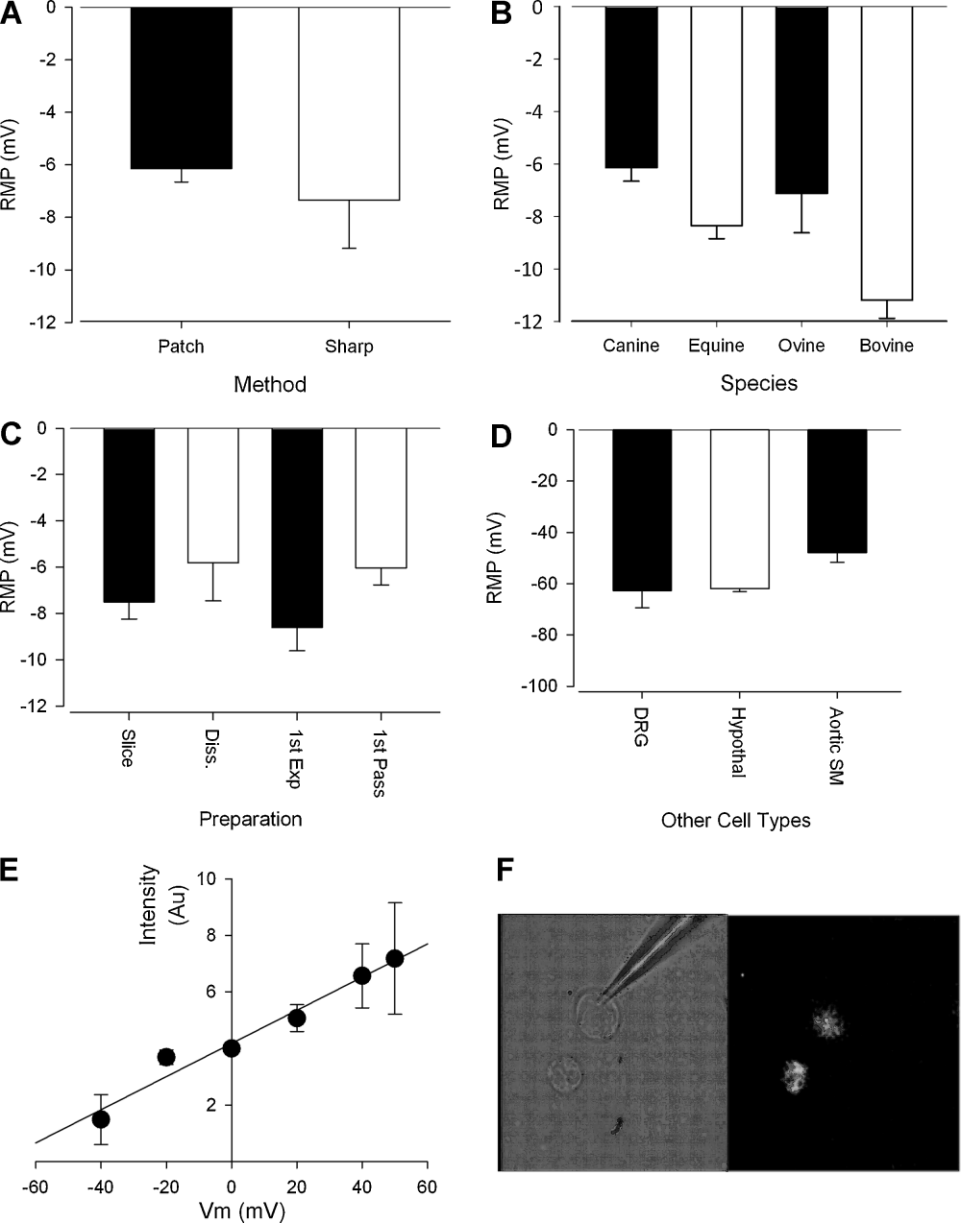
Freshly dissociated and primary cultured chondrocytes from a range of species exhibit relatively positive resting membrane potentials (RMP). A: RMPs measured in canine chondrocytes using patch clamp and sharp electrodes (n = 50, 19). B: RMP measured by the whole-cell patch-clamp technique in canine (n = 50, as A), equine (n = 9), ovine (n = 8), and bovine (n = 5) chondrocytes. C: RMP measured by sharp electrodes in cartilage slices (“slice,” n = 10) and whole-cell patch clamp from freshly dissociated canine chondrocytes (“diss.,” n = 5), from first expansion chondrocytes (“1st Exp,” n = 5) and canine chondrocytes following the first passage (“1st Pass,” n = 22). D: Control cell type demonstrating conventionally negative RMP; DRG, dissociated rat dorsal root ganglion neurones; hypothalamic, paraventricular nucleus pre-autonomic neurones; aortic SM, isolated smooth muscle cells from rat aorta. All patch-clamp experiments in this figure were performed with standard physiological intracellular and extracellular solutions, except the sharp electrode recording experiment in (A) where the extracellular solution was the same standard physiological saline, but the electrode was filled with 1 or 2 M KCl. E: Calibration curve for the voltage sensitive dye measurements, “AU” is arbitrary units of relative intensity. F: Two chondrocytes, one patched, one not patched, under visible (left part) and epifluorescence (right part) with oxonol VI dye, bar indicates 10 µm.

We measured the RMP of canine chondrocytes using both whole-cell patch clamp and sharp electrode recording. At temperatures of 24–25°C in physiological saline solution, we recorded values of −6.1 ± 0.5 mV, n = 50 with whole-cell patch clamp and −7.3 ± 1.8, n = 19 with sharp electrodes (Fig. 1A). The value measured by patch clamp was consistent across a range of large animal species (Fig. 1B). Studies have shown that these cells retain a substantially native chondrocyte phenotype for the first few passages in culture (Benya and Shaffer, 1982). We therefore compared RMP measured from chondrocytes in slices of cartilage (sharp electrodes −7.5 ± 0.7 mV, n = 10), freshly dissociated, first expansion and first passage chondrocytes (patch clamp). These were not significantly different to each other (Fig. 1C). As a control, we include RMP values recorded for other tissues on the same equipment; these all had RMP in the conventional significantly negative range (−48 to −64 mV) (Fig. 1D). To measure membrane potential from intact cells which had neither been impaled by a sharp electrode, or patch clamped, we also performed an optical dye study of the membrane potential (Fig. 1E,F). This gave a membrane potential value of −8.6 ± 8 mV, n = 14.

### The identity of the principal cation conductance open at rest

**Fig. 2:**
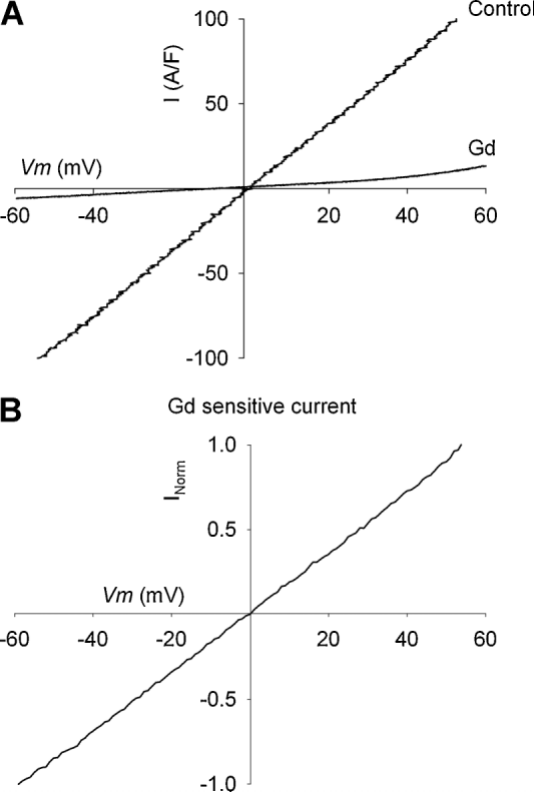
Patch clamp electrophysiology demonstrates the presence of gadolinium III sensitive whole-cell conductances. A: Whole-cell current ramps in “methanesulfonate solutions.” Command potential (V_m_) on the *x*-axis, current on the *y*-axis (normalized for cell size). Mean inhibition of whole-cell current was 80 ± 9%, n = 5, *P* ≤ 0.001. Control (vehicle) or in the presence of gadolinium III (Gd). B: The Gd sensitive current, normalized to that at −60 mV. The measured reversal potential of the difference current under these conditions was −1 ± 3 mV, n = 5.

Such a positive RMP in chondrocytes suggests that at rest the chondrocyte membrane is highly permeable to sodium and/or calcium ions (since these ions have equilibrium potentials above 0 mV). Having already investigated a number of potassium channels in the chondrocyte membrane (Mobasheri et al., 2005, 2007, 2010) we sought to identify cation channels with selectivity for sodium and/or calcium, at sufficient density to account for such a positive RMP. To investigate these conductances further, we switched to chloride and potassium-free “methanesulfonate solutions.” We compared whole-cell voltage ramps in the presence and absence of gadolinium (Gd), a widely used cation channel blocker (Fig. 2A). We found that the whole-cell current was sensitive to 100 µM Gd (Fig. 2A). The calculated Gd difference current is shown in Figure 2B.

**Fig. 3:**
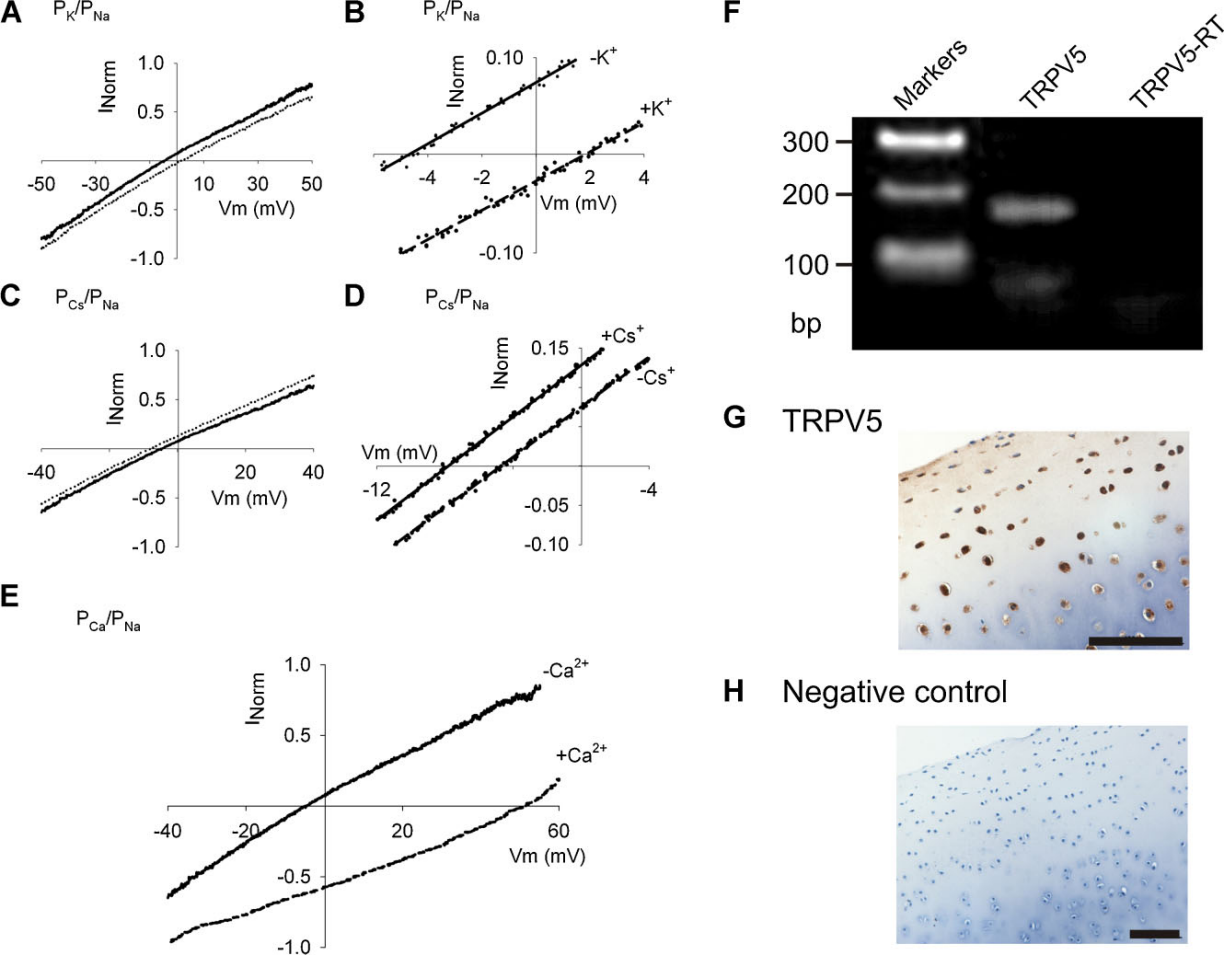
Gadolinium III difference current has high permeability to calcium ions. A,B: Gd sensitive current–voltage ramp with 150 mM external NaCl (solid line) or 150 mM external KCl (broken line). (B) shows the same data as (A), but magnified to show ΔV_rev_ more clearly. The permeability ratio P_K_/P_a_ (see text) was then calculated from Equation (1). C,D: Gd sensitive current–voltage ramp with 150 mM external NaCl (solid line) or 150 mM external CsCl (broken line). (D) shows the same data as (C), but magnified to show ΔV_rev_ more clearly. The permeability ratio P_Cs_/P_Na_ (see text) was then calculated from Equation (1). E: Current–voltage ramp with 150 mM external NaCl (solid line) or 30 mM CaCl_2_ and 105 mM NaCl (broken line). The permeability ratio P_Ca_/P_Na_ (see text) was then calculated from Equation (2). The full solutions for (A) to (E) are described in the Methods Section. F: RT-PCR was performed as described in the Methods Section. Specific primers for TRPV5 were used and mRNA (164 bp) product encoding TRPV5 was detected in extracts of first expansion chondrocytes. Omission of reverse transcriptase served as a negative control G, sections of full-depth canine articular cartilage were probed for channel expression by immunohistochemistry using polyclonal antibodies raised against TRPV5. H: Omission of primary antibody from the immunohistochemical procedure served as a negative control. Sections of cartilage were otherwise treated in exactly the same way during the immunohistochemical procedure except that the primary antibody was omitted. Positive immunoreactivity (brown staining) was observed in chondrocytes throughout normal cartilage. Bars in the main parts represent 100 µm.

**Fig. 4:**
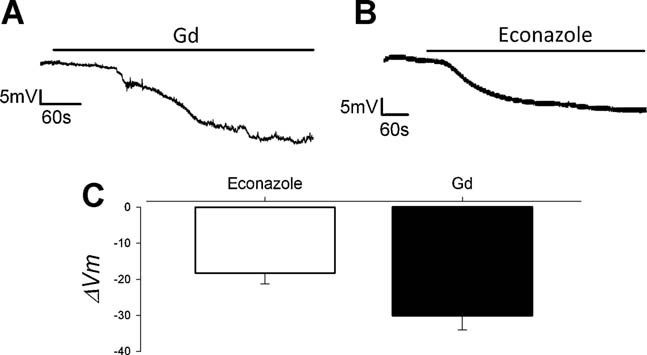
Gadolinium and econazole sensitive ion channels contribute to the RMP. Block of channels by Gd (A,C) or econazole (B,C) drive the membrane potential in a negative direction. Membrane potential record during the application of (A) 100 µM Gd, and (B) 10 µM econazole. C: Summary data for; 100 µM Gd (n = 8) and 10 µM econazole (n = 5). Each of these conditions significantly shifted the membrane potential.

Since Gd is well known to be a blocker of transient receptor potential channels including TRPV channels (Vennekens et al., 2001; Clapham, 2007; Alexander et al., 2008), we investigated if these channels are constitutively active in chondrocytes. There are several subtypes, however, and a useful way of distinguishing between them is by analysis of the conductance's permeability ratio, since many of the subtypes have distinct permeability profiles. To calculate the permeability ratio of the Gd difference we adapted the methods of Voets et al. (2002), and measured changes in reversal potentials of the Gd sensitive difference currents in extracellular solutions with different cation compositions (Fig. 3A–E). The permeabilities of Cs^+^ and K^+^ were not significantly different to that of Na^+^, but that of Ca^2+^ was significantly greater (P_K_/P_Na_ 1.02 ± 0.06, n = 10, P_Cs_/P_Na_ 1.2 ± 0.1, n = 9, P_Ca_/P_Na_ 78 ± 9, n = 5, *P* ≤ 0.0001). Since this permeability ratio is similar to that measured for TRPV5 (Vennekens et al., 2000; Owsianik et al., 2006) we investigated whether these cells contained TRPV5 mRNA by reverse-transcription PCR or expressed TRPV5 protein by immunohistochemistry. We clearly detected both TRPV5 mRNA and protein expression (Fig. 3F–H), confirming TRPV5 as a likely contributor to the Gd sensitive conductance. TRPV5 is known to be blocked by both Gd and econazole (Nilius et al., 2001) we investigated whether these compounds affected the RMP of chondrocytes (Fig. 4A,B). We found that both hyperpolarized the membrane, gadolinium by 30 ± 4 mV (n = 8, *P* ≤ 0.0005) and econazole by 18 ± 3 mV (n = 5, *P* ≤ 0.005; Fig. 4).

**Fig. 5:**
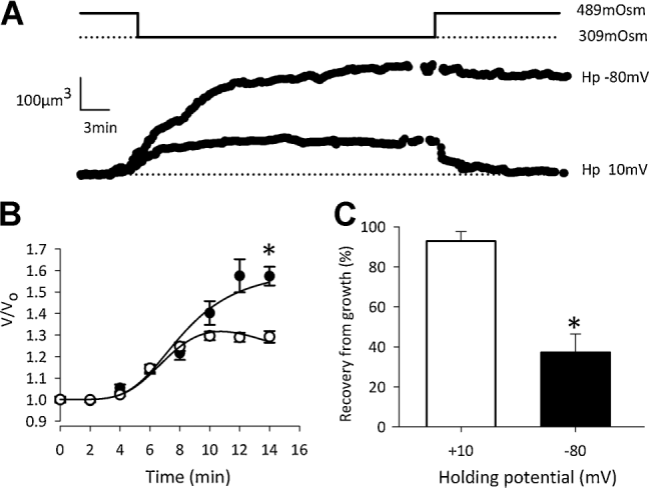
The positive RMP allows chondrocytes to more effectively regulate their volume. A: Change of cell volume with time during change of osmotic potential (where indicated). Cell volume was calculated at 30 sec intervals with cells voltage-clamped at either −80 or +10 mV with single sharp electrodes under switch clamp. B: Summary of a number of data such as that illustrated in (A) but with data sampled at 120 sec intervals. Cell swelling measured at 14 min was significantly greater when cells were held at −80 than +10 mV (*P* < 0.0005, n = 7 empty circles, 10 filled circles). The fitted line is one continuous fit to 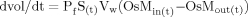 based on (Zhang et al., 1990; Preston et al., 1992), but including changes of intracellular osmolarity with time. The water permeability P_f_ = 10.2 × 10^−4^ cm sec^−1^, S_(t)_ the surface area and V_w_ the molar volume of water is 18 cm^3^ mol^−1^. Osmolarity inside and outside the cell at time t are OsM_in(t)_ and OsM_out(t)_, respectively. The only difference between the two fitted lines in (B) is the membrane potential. For a full explanation of the model see Appendix 1. C: On returning cells from the 309 mOsm to the 489 mOsm solution the size of cells clamped at +10 mV (n = 10) returned to near the pre-swell size whereas recovery at −80 mV (n = 12) was significantly less (*P* < 0.0005, n = 10, 12).

We hypothesized that the relatively positive RMP of chondrocytes would facilitate chondrocyte control of volume (see Appendix 1). In vivo chondrocytes are reported to experience osmolarities as high as 500 mOsm (Urban, 1994). On returning to lower osmolarity, chondrocytes swell (Bush and Hall, 2001a). To investigate the role of membrane potential in this process we used sharp-electrode voltage-clamp of chondrocytes with a switch-clamp amplifier. We controlled voltage and simultaneously calculated chondrocyte volume from measured 2D surface areas. Unlike conventional patch-clamp recording, which alters the intracellular environment of a cell, this method does not require alteration of the intracellular milieu. We measured cell volume continuously as the osmolarity of the extracellular bathing medium was reduced from 489 to 309 mOsm. Chondrocytes clamped at +10 mV and exposed to the reduced osmotic pressure increased in size by 129 ± 3%, n = 10 (Fig. 5A,B). This increase was reversible upon returning to the higher osmolarity solution (Fig. 5A,C). Strikingly, when this procedure was repeated with the membrane clamped to −80 mV, cell volume increased by 157 ± 4%, n = 12 (Fig. 5A,B) and the chondrocytes were no longer able to recover their volume (Fig. 5A,C).

## Discussion

We propose that a diverse chondrocyte channelome, including TRPV5, contributes to a relatively positive RMP in chondrocytes. Since this potential is significantly more positive than the equilibrium potential for potassium ions, it will allow the cell to efflux potassium ions rapidly enough to limit cell-volume increase under conditions of reduced osmolarity. This suggests that a relatively positive RMP is a biological adaptation to allow chondrocytes to survive the extreme osmotic challenges they routinely face.

### The positive RMP in chondrocytes

The relatively positive RMPs we report in the current paper are similar to one of the earliest report of chondrocytes' RMP (Wright et al., 1992) (sheep and human control chondrocytes; RMP: −10.6 and −12.4 mV, respectively). Since this time, a number of groups have observed more negative RMPs. These more negative RMPs include −46 mV on mouse chondrocytes (Clark et al., 2010), −41 mV on rabbit chondrocytes (Sugimoto et al., 1996), and −20 mV on a human cell line, OUMS-27 (Funabashi et al., 2010b). We have a number of possible explanations for this. We have used, over several years now, chondrocytes prepared from larger animals (Mobasheri et al., 2005, 2007, 2010). It is entirely possible that these larger animals have more positive RMP, since their joints experience greater forces than those of rodents (Huberti and Hayes, 1984; Clarke et al., 2001; Setterbo et al., 2009). Direct experimental comparison is difficult, however, due to the problems inherent in isolating a pure chondrocyte preparation from rabbits and small rodents. This is because these animals have cartilage depths as little as 55–300 µm (Stockwell, 1971; Frisbie et al., 2006; Ahern et al., 2009) and our isolation method involves manual shaving of cartilage from articular joints in a manner similar to the peeling of an apple. We feel we would, therefore, inevitably include other cell types in the extraction such as osteoclasts, osteoblasts, stromal cells, and cells of blood vessel origin. In the joints of the larger animals we used, cartilage depth was approximately 1 mm or more (from larger dogs to horses) and so we are confident of a pure chondrocyte preparation. Furthermore, our monolayer chondrocyte preparation reliably retains chondrocyte phenotype in terms of cells proliferation (Martin et al., 1999; Jakob et al., 2001; Schulze-Tanzil et al., 2004), secretion of collagen (type II) and chondrocyte specific proteoglycans for the first four passages in culture (Schulze-Tanzil et al., 2004). Isolated chondrocytes indeed exhibit similar volume regulating properties to in situ chondrocytes (Bush and Hall, 2001a,b), however, to ensure preservation of the chondrocyte phenotype, we used freshly dissociated, first expansion and first passage chondrocytes only. It is notable that we observe the relatively positive RMP even in slices of cartilage or in first passage chondrocytes.

### The identity of the principal cation conductances open at rest

In previous reports we have focused on potassium conductances in chondrocytes (Mobasheri et al., 2005, 2007, 2010). In this study, however, we focused on non-potassium cation channels open at rest. Our whole-cell current–voltage ramp protocols clearly demonstrate that the majority of the total resting (non-potassium) current is generated by a Gd sensitive conductance. It is likely that the Gd sensitive conductance is comprised of more than one type of channel, however, our permeability data suggest that TRPV5 dominates. TRPV5 is one of the few ion channel conductances with little selectivity between Cs^+^, Na^+^, and K^+^ ions, but high permeability to Ca^2+^ ions (Vennekens et al., 2000; Owsianik et al., 2006; Alexander et al., 2008). The presence of both TRPV5 mRNA and TRPV5 protein by RT-PCR and immunohistochemistry serve to further support the notion that these cells express TRPV5. In addition, econazole, a relatively selective inhibitor of TRPV5 (Nilius et al., 2001) hyperpolarized the membrane in a similar manner to Gd itself. That Gd hyperpolarizes the chondrocyte by more than the econazole implies that Gd may additionally inhibit other conductances in the cell, and these will be the subject of future investigations.

### The physiological role of the RMP in chondrocytes

It seems likely that the unusual electrical properties of chondrocytes must confer a biological advantage to these cells. We propose that this advantage is the ability to withstand changes in osmotic potential. This follows because the rate and degree of cell swelling during osmotic shock is counteracted by the release of ions, particularly K^+^ and concomitant reduction in osmotic drive for water entry (Hoffmann and Dunham, 1995; Hoffmann et al., 2009). One would expect that the predicted loss of potassium ions would be matched by an equal number of anions (e.g., chloride), reducing the absolute loss of potassium ions. Several different chondrocyte potassium channels have been proposed to open in response to membrane stretch and conduct these potassium ions (Hall et al., 1996; Martina et al., 1997; Mobasheri et al., 2010). From this, we predicted that cell swelling would be much less at a positive RMP than it would be at substantially negative membrane potentials. This prediction follows from the fact that the driving force (RMP − E_k_) for potassium ion efflux is much greater at more positive membrane potentials. The relationship is given by:


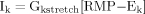
(4)

where I_K_ is the potassium current, G_kstretch_ is the potassium conductance, and E_K_ the equilibrium potential for potassium ions. We tested this prediction by experiment. One approach to this experimental design would be to decrease osmolarity from approximately 300 mOsm to, for example, 200 mOsm, however, it is generally accepted that this is well outside the chondrocyte's normal environmental range (Urban, 1994). Therefore, in order to make this experiment as physiologically relevant as possible, we measured volume increases when decreasing osmolarity from a relatively high (approximately 490 mOsm), to a physiological minimum for cartilage (approximately 320 mOsm, Urban, 1994). We found that positive RMPs significantly reduced the volume increase when cells were exposed to reduced osmotic pressure. At positive membrane potentials this increase in volume was very similar to that previously measured in unclamped chondrocytes in culture or in situ (Bush and Hall, 2001b).

Interestingly, our data show that at very negative membrane potentials, chondrocytes appeared unable to decrease their volume when exposed to the higher osmotic potential solution, that is, cell shrinkage was slower, or non-existent. This could also be a consequence of the principle encapsulated by Equation (4), or it could be that cells suffered damage from the osmotic challenge. The relationship between osmotic pressure and physical pressure is an important one for the chondrocyte, since it is believed that increasing compressive loads on joints leads to increases in osmotic pressure (Mow et al., 1992; Urban, 1994). The ability of chondrocytes to withstand osmotic pressure changes is therefore coupled to their ability to withstand mechanical pressure. Indeed it has been shown that cells are more susceptible to physical damage at reduced osmolarities (Bush et al., 2005). Furthermore, chondrocytes from osteoarthritic cartilage have been shown to exhibit poor recovery from cell volume increases (Jones et al., 1999) and it has been suggested that inappropriate increases in chondrocyte volume may contribute to the progression of osteoarthritis (Bush and Hall, 2003). Maintenance of the relatively positive membrane potential may therefore be important for the function and survival of healthy chondrocytes in vivo.

## Appendix 1

### Dependence of Osmotic Swelling Upon Membrane Potential

To simulate the swelling of cells at different membrane potentials we used a variation of the equation from Zhang et al. (1990; Preston et al., 1992), with substitutions to allow for time variable osmolarities

(5)

where P_f_ is the water permeability of the cell (cm sec^−1^), SA_(t)_ the surface area and the molar volume of water (V_w_) is 18 cm^3^ mol^−1^. The extracellular osmolarity (Osm_out_) is assumed to not change with time, but the osmolarity inside the cell at time t (Osm_in(t)_) is given by:

(6)

where Osmol_in_ is the number of moles of intracellular solute (osmol), not the osmolarity. This changes as potassium ions efflux and so the rate of change in the moles of ions within the cell is equivalent to:

(7)

where e is the elementary charge and N_A_ is the Avogadro's number. G_Kstretch_ is the potassium conductance opened by stretch, this is likely to be composed of calcium activated potassium channels (Wright et al., 1992; Martina et al., 1997; Mobasheri et al., 2010). We then used the simplest relationship for activation of this conductance at time t; we assumed that:

(8)

*Stretch* was calculated simply as the fractional change in surface area.  (the total stretch activated potassium conductance, 660 pS) and A (a constant of proportionality, 10^−4^ sec^−1^) were both obtained by fitting Equation (5) (by numerical integration) to the volume data by minimizing *χ*^2^. Additionally, for the cell to maintain electroneutrality the K^+^ efflux is likely to be matched by an anionic efflux. All our simulations also included a routine to account for the time-dependent change of extracellular bath solution. This was relatively slow in some experiments (approximately 6 min), since sharp electrodes dislodge more readily than patch electrodes and we observed cells to be rather fragile when maintained at very hyperpolarized potentials. The only parameter changed between the simulations at −80 and +10 mV is the RMP parameter in Equation (7).

## Abbreviations

RMP: resting membrane potential
TRP: transient receptor potential channel
TRPV: vanilloid type transient receptor potential channel
TRPV5: vanilloid type transient receptor potential channel
